# The Role of Lignin Molecular Weight on Activated Carbon Pore Structure

**DOI:** 10.3390/molecules29163879

**Published:** 2024-08-16

**Authors:** Chengjun Wu, Junhuan Ding, Graham W. Tindall, Zachariah A. Pittman, Mark C. Thies, Mark E. Roberts

**Affiliations:** Department of Chemical and Biomolecular Engineering, Clemson University, Clemson, SC 29634, USA

**Keywords:** activated carbon, lignin, lignin molecular weight, bioproducts, lignocellulosic, biorefinery

## Abstract

Over the past decade, the production of biofuels from lignocellulosic biomass has steadily increased to offset the use of fuels from petroleum. To make biofuels cost-competitive, however, it is necessary to add value to the “ligno-” components (up to 30% by mass) of the biomass. The properties of lignin, in terms of molecular weight (MW), chemical functionality, and mineral impurities often vary from biomass source and biorefinery process, resulting in a challenging precursor for product development. Activated carbon (AC) is a feasible target for the lignin-rich byproduct streams because it can be made from nearly any biomass, and it has a market capacity large enough to use much of the lignin generated from the biorefineries. However, it is not known how the variability in the lignin affects the key properties of AC, because, until now, they could not be well controlled. In this work, various fractions of ultraclean (<0.6% ash) lignin are created with refined MW distributions using Aqueous Lignin Purification using Hot Agents (ALPHA) and used as precursors for AC. AC is synthesized via zinc chloride activation and characterized for pore structure and adsorption capacity. We show that AC surface area and the adsorption capacity increase when using lignin with increasing MW, and, furthermore, that reducing the mineral content of lignin can significantly enhance the AC properties. The surface area of the AC from the highest MW lignin can reach ~1830 m^2^/g (absorption capacity). Furthermore, single step activation carbonization using zinc chloride allows for minimal carbon burn off (<30%), capturing most of the lignin carbon compared to traditional burn off methods in biorefineries for heat generation.

## 1. Introduction

Further development and utilization of energy crops for the purpose of producing lignocellulosic biofuels will result in a dramatic reduction in atmosphere CO_2_ levels by replacing or substituting fuels derived from petroleum. The effect of CO_2_ reduction can be compounded when the “ligno-” component of the biomass can be converted into a solid product rather than burning it for its heating value, which thereby results in a net-negative CO_2_ output. By 2030, the global biofuels market is anticipated to expand significantly, reaching a value of USD 176.5 billion [1]. Instead of relying on expensive and edible biomass as feedstock, using lignocellulosic biomass is a better option for producing biofuels. This type of biomass is readily available in nature and is much more cost-effective [2,3,4,5]. During the biofuel production process, biorefineries extract cellulose and hemicellulose to produce biofuels, while separating lignin as a byproduct [4,5,6]. Currently, most of the separated lignin from biorefineries is burned as a low-value fuel for heat and electricity generation, thereby returning the carbon back to the environment. A similar situation occurs in paper mills, where cellulose is used to produce paper and lignin is burned. In all, nearly 100 million tons of lignin are separated annually from biorefineries and paper mills, while 98% of them are combusted for energy recovery [7,8].

Lignin, which comprises up to 30% of the mass of the lignocellulosic biomass [9,10], is the most abundant natural biopolymer that is rich in aromatic functionality. Instead of combusting lignin for heat or electricity generation, it can be used to produce chemicals and incorporated into various materials, such as hydrogels [11], polyurethane foams [12], epoxy resin [13], and phenolic powder resin [13,14]. Moreover, its high carbon content makes it suitable for carbon-rich products, including carbon fiber [15] and activated carbon [16]. Notably, lignin is a natural polymer, and its use as an alternative to replace petrochemical precursors for these products can significantly reduce the need for fossil fuel. However, the application of lignin is sometimes less than perfect. The main issue is that the variability in the properties of lignin, such as impurity content and heterogeneous molecular weight (MW), is inevitable, as they arise from different natural environments where the lignin originates. These inconsistencies pose challenges to the industrial application of lignin. For instance, carbon fiber production requires very clean lignin precursors with high molecular weight, otherwise, its mechanical performance will be poor [17].

Activated carbon (AC), also called activated charcoal, is a kind of processed carbon characterized by high specific surface area and controllable porosity [16]. It can be produced from carbonaceous source materials such as wood [18], coal [19], biomass [20,21,22,23], or petroleum pitch [24] through physical or chemical activation. Physical activation, which is a two-step process, involves carbonizing the material and then activating it at high temperatures using steam, carbon dioxide, or air as the activating agent [16], resulting in a wider pore size distribution but higher burn off and lower yield. On the other hand, chemical activation is a one-step process that simultaneously activates and carbonizes the carbonaceous material at a low temperature with a chemical activating agent like zinc chloride, potassium hydroxide, or phosphoric acid. This method leads to a narrower pore size distribution, a lower burn off, and generally higher yields compared to physical activation.

The porous structure of AC gives rise to high specific surface areas and particularly high adsorption capacities, making it widely used in water/gas purification [25,26,27,28], food/beverage processing [29], heterogeneous catalysis [30], and electronics [31,32,33,34]. The global AC market size was estimated at USD 4.92 billion in 2023 and is projected to grow at a compound annual growth rate of 6.0% from 2024 to 2030 [35]. This large market volume is important because of the mass quantities of available lignin byproducts. In addition to the inherent demand for AC for the applications noted above, converting lignin from energy crops into solid carbon serves a dual purpose of carbon capture and storage. Theoretically, the amount of AC that can be produced from lignin indicates the amount of carbon that can be captured and stored. However, that is not all. An increase in the supply of AC, specifically for the purpose of water purification, will result in lower-cost clean water. Since AC does not have strict limitations on its precursor quality, theoretically, any lignin can be carbonized and activated. Lignin with a high impurity content and varying molecular weight, which cannot be utilized in other applications, can be converted into AC. Such flexibility allows lignin with inconsistent properties to be utilized in AC production, expanding its potential uses across various industries.

The general goal of synthesizing AC is to achieve the highest surface area at the lowest cost (higher AC yield). To achieve this, particularly with a biopolymer like lignin, which inherently exhibits inconsistent properties, it is necessary to understand the relationship between lignin properties and the key performance metrics of AC. Previous studies on using lignin as a precursor for AC have shown a wide range of properties, with surface area calculations from <10 to 2753 m^2^/g, substantial variations in overall product yields, and differences in pore size and distribution [36]. The discrepancies in results arise because of variations in the activation processes (either physical or chemical) and lignin molecular properties. Until recently, previous studies have typically focused on evaluating the effects of activation agents and/or carbonization conditions on AC properties, and they rarely consider how the properties of lignin affect the resulting activated carbon.

**Figure 1:**
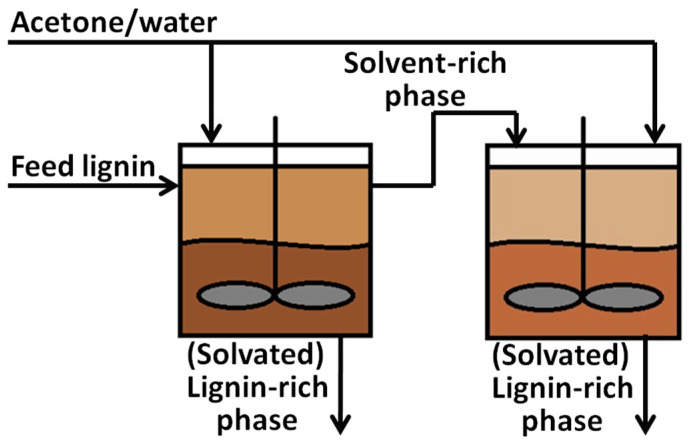
Aqueous Lignin Purification using Hot Agents (ALPHA) Process for fractionation of lignin.

In our previous work, we presented clear correlations between the composition of lignocellulosic biomass—lignin, cellulose/hemicellulose, and ash content—and the AC surface area, pore width, pore distribution, and carbon fractional conversion [37]. Despite these findings, it is less clear how lignin and its properties affect the resulting activated carbon, because controlling lignin properties at the molecular level is challenging. In response to the difficulty in using lignin as a precursor for carbon-based products, the Aqueous Lignin Purification using Hot Agents (ALPHA) process [15,38,39] (Figure 1) has been developed to purify and fractionate lignin based on its molecular properties. The ALPHA process involves mixing the lignin sample with an organic solvent at an elevated temperature to create a liquid–liquid equilibrium, resulting in the formation of a lignin-rich phase and a solvent-rich phase. In this process, higher MW lignin is generally directed to the lignin-rich phase, while lower MW goes to the solvent-rich phase. By adjusting the operation conditions, different ultraclean lignin fractions with refined MWs and distributions can be tailored during the ALPHA process. This method enables some degree of control over the lignin MW.

Here, we investigate the relationship between lignin MW and mineral content on the key performance metrics of AC, including surface area, pore volume, pore size, and carbon fractional conversion. Pure lignin samples with different MWs and narrow MW distributions were isolated from a commercial Kraft lignin feedstock using the ALPHA process [15,38,39]. AC was synthesized from these lignin samples using a ZnCl_2_ activation process with relatively low-temperature carbonization. We found that the surface area of AC is correlated with lignin, and pore enlargement is promoted in the lignin sample with lower MW. This is because lignin from biorefineries and paper mills typically contains considerable amount of ash content, and the effect of lignin MW on the AC product is often coupled with the effect of ash content when directly using feedstock lignin. Therefore, we also demonstrate how the lignin ash content affects AC properties by controlling for lignin MW. The surface area and total pore volume of AC are lower when synthesized from lignin samples with a higher ash content, and the higher ash content also promotes pore enlargement.

## 2. Results and Discussion

### 2.1. Lignin Samples for Activated Carbon

A commercial lignin, named Biochoice^®^ lignin, was used as the feed lignin (LF) in this work to study the relationship between the molecular weight (MW) and the ash content of lignin and the properties of AC. This lignin is classified as a sugar-free, low-ash lignin, produced from Southern Pine trees via the Kraft process. The Aqueous Lignin Purification using Hot Agents (ALPHA) process, described in greater detail elsewhere [15,38,39], was used to fractionate the LF to obtain clean lignin with different MWs by manipulating the organic solvent/water ratio and temperature. Typically, during the ALPHA process, the higher MW lignin goes to the lignin-rich phase, while the lower MW lignin and ash remain in the solvent-rich phase. By separating the lignin-rich phase from the solvent-rich phase, a clean, high-MW lignin can be obtained. The solvent-rich phase can then be subject to a second stage of ALPHA processing to extract a clean, low-MW lignin. Finally, the solvent-rich phase from the second stage containing impurities, such as metals and salts, along with very-low-MW lignin, is discarded.

**Figure 2:**
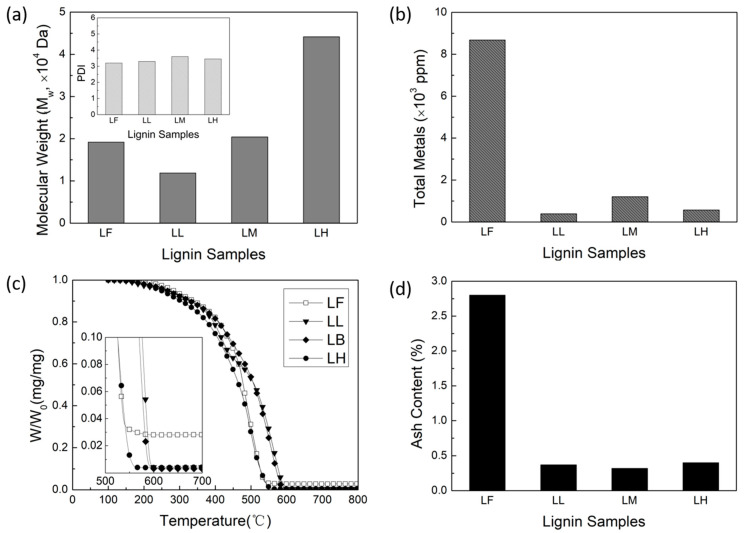
(**a**) Weight-average molecular weight (M_w_), polydispersity index (PDI, inset), (**b**) metal content, (**c**) thermogravimetric (TG) curve, and (**d**) ash content of lignin sample.

Figure 2a shows the MW of the lignin samples, as determined from size exclusion chromatography, followed by multi-angle light scattering (SEC-MALS), as well as the polydispersity index (PDI) in the inset. Through the ALPHA process, we were successful in producing a range of lignin samples with different MWs. Compared to the feed lignin (LF), which had a weight-average MW (M_w_) of 19,200 Da, the resulting lignin samples spanned a range of M_w_ from 11,900 Da to 44,200 Da. Specifically, the following three distinct lignin samples were obtained: the low-MW lignin (LL) with an M_w_ of 11,900 Da, the medium-MW lignin (LM) with an M_w_ of 20,400 Da, and the high-MW lignin (LH) with an M_w_ of 44,200 Da. Furthermore, the PDI values of the three ALPHA-processed lignin samples are similar to the PDI values of the LF, indicating that the ALPHA process does not apparently alter the distribution of lignin MW. These three samples all have narrow MW distributions.

The metal content (wt%) of the lignin samples was determined by Inductively Coupled Plasma Atomic Emission Spectroscopy (ICP-AES) and is shown in Figure 2b. The primary metal in the LF is sodium, which came from the delignification process (Kraft process for Kraft lignin). After the ALPHA process, nearly 90% of the metals are reduced, resulting in LL, LM, and LH all exhibiting very low metal contents lower than 1200 ppm. Figure 2c displays thermogravimetric analysis (TGA) profiles of the LF and the three ALPHA-processed lignin samples. The ash content of the lignin samples, determined as the residue remaining after TGA at 700 °C, is presented in Figure 2d. Similar to the metal content, ash in the lignin is much reduced after the ALPHA process. Compared to the ash content of 2.8% in LF, all of the ash contents in the three ALPHA-processed lignin samples are lower than 0.6%.

### 2.2. Activated Carbon Yield and Fractional Conversion

**Figure 3:**
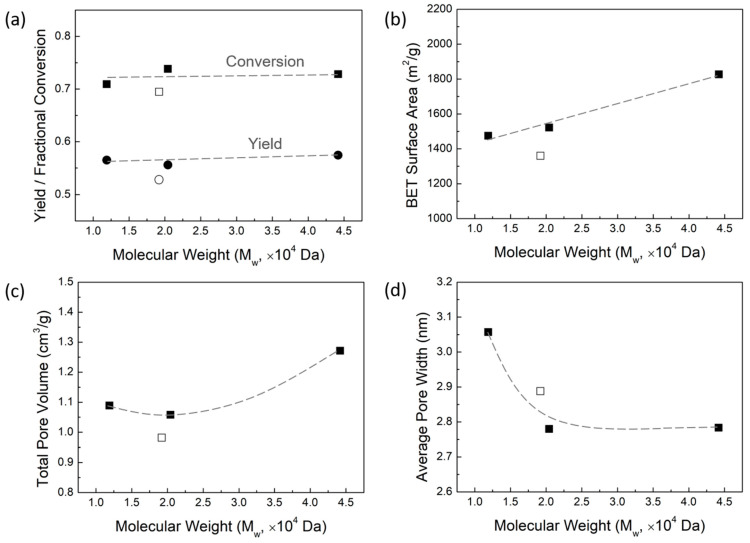
(**a**) Overall yield (mass of activated carbon (AC)/mass of lignin) and carbon fractional conversion (mass of C in AC/mass of C in lignin) as a function of the molecular weight (MW) of lignin. (**b**) Surface area, (**c**) total pore volume, and (**d**) average pore width of AC as a function of lignin MW.

Taking into account the goal of saving energy and achieving the highest possible overall AC yield, AC was synthesized from the LF and the three ALPHA-processed lignin samples using constant ZnCl_2_ activation with low-temperature carbonization conditions. Chemical activation using ZnCl_2_ was chosen because the previous literature has shown that high carbon yields can be obtained, along with high surface areas, using low-temperature carbonization [40]. Figure 3a shows the overall yield of AC and carbon fractional conversion plotted as a function of the MW of lignin. The overall yield of AC is the amount of AC obtained from the lignin, defined as the mass of AC divided by the mass of the starting lignin sample. The carbon fractional conversion is the proportion of carbon in lignin that ends up in AC, defined as the mass of carbon in AC divided by the mass of carbon in lignin. In Figure 3a, the overall AC yields and the carbon fractional conversions of the three ALPHA-processed lignin samples are fairly high, all being higher than 50% and 70%, respectivley. This is due to the fact that ZnCl_2_, as an activating agent, inhibits the gasification and tar formation in the carbonaceous material of the lignin [41,42,43]. At the same time, the overall AC yields and the carbon fractional conversions of the three ALPHA-processed lignin samples are relatively consistent, ranging between 55–58% and 71–74%, respectively. This suggests that variations in lignin MW have little effect on the overall AC yield and the carbon fractional conversion, which corresponds to the results of our previous work showing that the clean lignin samples have the same carbon fractional conversions [37]. Compared to the ALPHA-processed samples, AC derived from the LF exhibits a lower overall AC yield and carbon fractional conversion. Considering that the LF has a high ash content (2.7%), the lower overall AC yield and carbon fractional conversion indicate that ash negatively impacts the conversion from lignin to AC. One possible explanation for this phenomenon could be that the ash, during the preparation of AC, functions as a catalyst, accelerating the burn off of the lignin carbon [44,45].

### 2.3. Pore Structure of Activated Carbon

#### 2.3.1. Effect of MW of Lignin on Pore Properties

Figure 3 displays the surface area, pore volume, and average pore width of AC as a function of MW of lignin in panel b, c, and d, respectively. The surface area of AC is interpreted from the nitrogen (N_2_) adsorption isotherm obtained at 77 K, based on the Brunauer–Emmett–Teller (BET) theory. The total pore volume is determined based on the N_2_ adsorption amount, expressed in liquid form at 77 K and a relative pressure (P/P_0_) of approximately 0.95. The average pore width is calculated from the total pore volume and the surface area of AC using the Gurwitch rule. As the lignin MW increases, the surface area of AC has a linear growth, ranging from 1430 to 1830 m^2^/g for the lignin sample with an MW value varying from 12,000 to 44,000 Da. The total pore volume of AC also increases with the increasing MW, with the lignin of the highest MW having the highest total pore volume of 1.27 cm^3^/g. Notably, the LL sample and the LM sample have comparable total pore volumes. As shown in Figure 3d, the average pore width of AC is much greater in the lignin samples with low MW, while the average pore width of AC from the medium- and high-MW lignin samples remains relatively consistent.

Generally, during low-temperature carbonization with ZnCl_2_ activation of the lignocellulosic biomass, the evolution of the porous structure of AC is driven by the release of functional groups from the carbon skeleton as volatiles, leaving vacancies behind [44,46,47,48]. This process is governed by the following two primary factors: the generation of new pores or the enlargement of existing pores. Once a new pore is formed, whether the chemical activation agent (ZnCl_2_) continues to act on this pore to enlarge its size or acts elsewhere to create new pores largely depends on the sample properties of the lignocellulosic biomass. Typically, these two processes occur simultaneously, only to different degrees. The pore properties of AC are essentially a reflection of these two processes. Our previous study discussed that an evolution of porous structure favoring pore creation typically results in a higher surface area [37]. According to the pore properties of AC in Figure 3, it can be observed that, when the lignin is in high- and medium-MW range (approximately greater than 2 × 10^4^ Da), a higher MW of lignin is more conducive to new pore creation, thus obtaining a higher specific surface area. However, when the lignin is in the low-MW range (approximately less than 2 × 10^4^ Da), a decrease in lignin MW progressively enhances pore enlargement in the existing pores. This leads to the AC derived from LL exhibiting a larger average pore width.

Despite the LF and LM samples having similar lignin MWs, the AC derived from the LF exhibits a lower surface area and pore volume, but a higher average pore width compared to the AC from the LM. This disparity could potentially be attributed to the high ash content of the LF. Specifically, the ash might be reducing the surface area and total pore volume of the AC, while simultaneously enlarging the pores, as indicated by the higher average pore width in AC derived from LF. As reported in the literature [49], a high ash content can obstruct the entry of chemical activating agents into lignin particles, thereby reducing the contact area between the activation reagent and the lignin. Moreover, ash content can also block the channel for the off-gases releasing, which is unfavorable to the development of pores. Therefore, the surface area and total pore volume of the higher ash content sample are lower. For the higher average pore width, this is because the ash can also impede the penetration of ZnCl_2_ into the lignin particle, causing pore enlargement of the exterior pores. Furthermore, ash weakens the intermolecular forces between the lignin molecules, making it easier for pores to enlarge. Metals, such as Na, can act as catalysts during the carbonization process, accelerating the thermal decomposition of lignin and contributing to the enlargement of the existing pores [44,45].

#### 2.3.2. Effect of MW of Lignin on Pore Size Distribution

**Figure 4:**
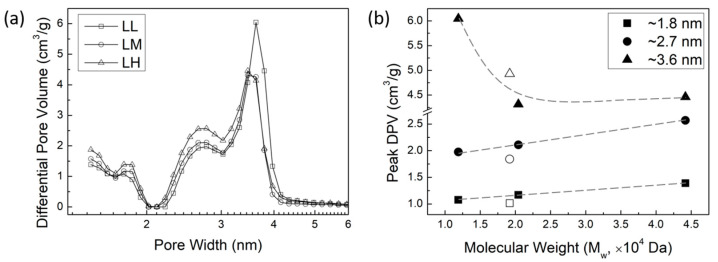
(**a**) Pore size distributions in AC from lignin samples (represented as differential pore volumes at each pore width) with varying MW of lignin. (**b**) Peak values of differential pore volume as a function of MW of lignin at pore widths of ~1.8 nm, ~2.6 nm, and ~3.6 nm.

To investigate how lignin MW affects pore size distribution of AC, the N_2_ desorption data were analyzed using the density functional theory (DFT). Figure 4a shows the pore size distribution of AC of the ALPHA-processed lignin with varying MW, represented as differential pore volumes at each pore width. Three distinct peaks in pore size distribution are observed at approximately 1.8 nm, 2.7 nm, and 3.6 nm, representing the primary pore widths within the AC. In other words, a peak value in differential pore volume around a specific pore width indicates that the AC contains more pores at this size. As the MW of the lignin increases, the pore volume of the micropores (<2.0 nm) and small mesopores (~2.7 nm) increases. However, the pore volume of the larger mesopores exhibits a different trend. The peak shapes observed for the larger mesopores around 3.6 nm of the LM and LH samples are similar, but the LL sample displays a much higher and broader peak compared to the higher MW samples.

Figure 4b illustrates peak values in differential pore volume vs. MW of lignin. It shows a linear increase in the differential pore volume of micropores (<2.0 nm) and smaller mesopores (~2.7 nm) as the MW of lignin increases. Considering that the micropores and smaller mesopores contribute significantly to the surface area and total pore volume of the AC [37], the increase in the surface area and the total pore volume of AC observed in Figure 3b,c can be attributed to the development of these micropores and smaller mesopores. Moreover, the trend in the differential pore volume peak of larger mesopores (~3.6 nm) mirrors the trend of the average pore width with increasing lignin MW, as observed in Figure 3d. The notably larger average pore width of the LL sample in Figure 3d is due to the higher pore volume of larger mesopores, as indicated by the broader and higher peak around 3.6 nm.

From the pore size distribution in AC from lignin samples with varying MW, we can deduce how lignin MW affects the pore properties of AC. When the MW of lignin is in a medium- and high-MW range (greater than 2 × 10^4^ Da), the development of micropores and smaller mesopores remains in a certain balance with pore enlargement, leading to a consistent average pore width in AC (~2.78 nm, as shown in Figure 3d). When the MW of lignin is in a low-MW range (less than 2 × 10^4^ Da), the pore enlargement significantly enhances with the decrease in lignin MW. This enhanced pore enlargement does not affect the linear growth of the surface area of AC with lignin MW, but it results in a relatively higher pore volume in the LL sample, causing the pore volume of AC to not linearly increase with the MW of lignin like the surface area does (shown in Figure 3c).

In fact, the underlying mechanism of the development of micropores and smaller mesopores is the generation of new pores. Our previous study found that, once a new pore forms, whether the chemical activation agent (ZnCl_2_) continues to act on this pore to enlarge it or act elsewhere to create new pores depends on the reactivity of the lignocellulosic biomass sample [37]. If the sample is more reactive, then the process of generating new pores is more pronounced, while, if the sample is less reactive, the enlargement of the existing pores becomes more significant. This is because once a pore forms and disrupts the aromatic structure, it becomes more favorable for reactions to occur within this disrupted carbon system than to initiate the formation of new pores. As the MW of lignin increases, the more pronounced impact of the generation of new pores suggests that the higher MW lignin is more reactive. However, when dealing with the lignin of lower MW, the significant role of pore enlargement implies that lower MW lignin is less reactive.

Considering that the oxygen-containing groups and aliphatic structures in lignocellulosic biomass exhibit more reactivity than non-oxygen-containing groups and aromatic structures [37], the pore enlargement observed in the lower molecular weight lignin could be attributed to the following factors: (1) decreased oxygen content, (2) reduced presence of aliphatic carbons, or perhaps a combination of both factors. During the synthesis of AC, oxygen-containing groups, aliphatic carbons, or aliphatic oxygen-containing groups in higher MW lignin are more easily converted to volatiles and released in the off-gas, facilitating the creation of new (small) pores. While lower MW lignin is relatively less reactive, once a pore is created and the aromatic structure is disrupted, reactions involving the disrupted carbon system become more favorable compared to generating new pores. This results in the enlargement of existing pores rather than the formation of new pores. Furthermore, the literature has also reported that polymers with lower MW tend to have closer contact with each other, thus yielding higher density [50]. Lower MW lignin may possess a stacked aromatic structure, which impedes the penetration of chemical-activating agents into the lignin particles. Therefore, the pores created on the outer surface of the lignin particles experience enlargement in the presence of excess ZnCl_2_.

**Figure 5:**
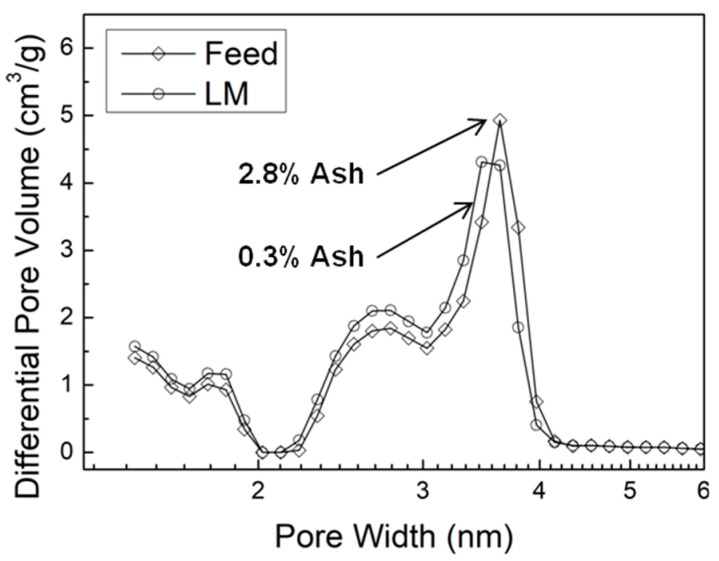
Pore size distributions in AC from lignin feedstock (LF) and ALPHA-processed medium-MW lignin sample (LM).

Because LF, the original lignin feed, and LM have similar MW values, and the ash content in the ALPHA-processed LM sample is much lower, it is possible to determine the impact of ash content on AC pore properties. The pore size distributions of AC from the LF sample and the LM sample are shown in Figure 5. The AC derived from the LF (higher ash content) exhibits a smaller volume of micropores and small mesopores (2.7 nm), as indicated by the lower peak values around 1.8 nm and 2.7 nm, respectively. However, they exhibit a larger volume of large mesopores (3.6 nm), signified by the higher peak value around 3.6 nm, compared to the LL sample that has an extremely low ash content. This observation is consistent with the previously discussed conclusion, explaining why the AC derived from LF exhibits a higher average pore width, as shown in Figure 3d. In other words, the presence of ash facilitates the enlargement of existing pores more readily than the formation of new pores, as discussed above.

### 2.4. Aqueous Adsorption Capacity of Activated Carbon

**Figure 6:**
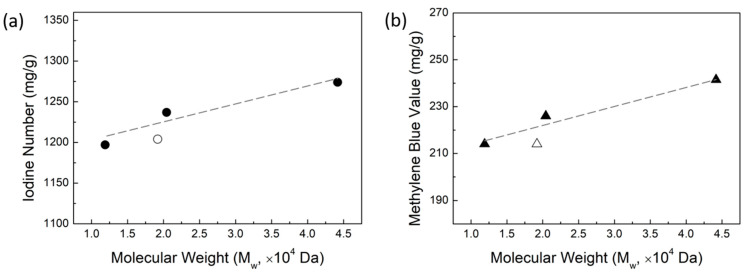
(**a**) Iodine number and (**b**) methylene blue value of AC as a function of lignin MW.

The aqueous adsorption capacity of AC is often evaluated using either the iodine number or methylene blue (MEB) value, which represent the amount of each chemical that can be absorbed on a given carbon surface. Figure 6 shows the results for the iodine and MEB adsorption measurements in panels a and b, respectively. The adsorption values are reported as mg of species absorbed per g of AC. Regardless of the size of the molecular adsorbate (iodine is small and MEB is fairly large), the aqueous adsorption capacity of the AC increases as the lignin MW increases in a manner consistent with the change in surface area with lignin MW. This trend indicates that the aqueous adsorption capacity of AC is determined by the surface area of the AC, rather than by the total pore volume.

## 3. Materials and Methods

### 3.1. Lignin Sample Preparation

The lignin feedstock (LF) was obtained from a commercial source, named Biochoice^®^ lignin, which is supplied by Domtar Corp (Plymouth, NC, USA). The LF was fractionated through the ALPHA (Aqueous Lignin Purification using Hot Agents) process [15,38,39]. In all cases, a 50 mL reactor (model 4593, Parr Instrument, Moline, IL, USA) was used for ALPHA processing, and ~10 g of the LF was charged into the reactor along with ~30 g of solvent. The mixture was stirred and heated to a specific temperature, allowing it to mix for at least 15 min. After the mixing time, the reactor was opened, revealing a liquid lignin-rich (LR) and solvent-rich (SR) phase. The LR phase adhered to the impeller of the reactor and was then collected into a sample pan, while the SR phase was decanted into a separate pan. Both phases were dried to remove the solvent and ground into a powder with a mortar and pestle. For all fractions, acetone/water mixtures were used as a solvent. All further references to compositions will be denoted on a weight basis.

Three lignin fractions of different molecular weights (MW) were obtained independently from the ALPHA process. They are a high-MW lignin fraction (LH), a medium-MW lignin fraction (LM), and a low-MW lignin fraction (LL).

LH was obtained from the LR phase using a solvent composition of 5:5 (acetone to water), a temperature of 45 °C, and a solvent-to-lignin ratio of 3:1. LM was also obtained from the LR phase, utilizing a solvent composition of 3:7, a temperature of 45 °C, and a solvent-to-lignin ratio of 3:1. The low-MW lignin fraction (LL) was acquired via a two-stage ALPHA process. Initially, an SR phase was isolated using 50% acetone at 45 °C. This SR phase was then dried to a powder to recover a low-MW fraction. This fraction was charged to stage 2, where it was contacted with a 40/60 *w*/*w* acetone/water solution with the LR phase, then recovered as the final product. This second stage was operated at 25 °C. Lignin yields from the LF of the high-MW fraction, the medium-MW fraction, and the low-MW fraction were controlled at 14%, 84%, and 50%, respectively, at their own individual runs.

### 3.2. Lignin Characterization

The molecular weight (MW) of the lignin was determined using SEC-MALS (size exclusion chromatography, followed by multi-angle light scattering). The lignin was first dissolved at a nominal concentration of 3 mg/mL in a solution of 0.05 M LiBr in dimethyl formamide (DMF). The mixture was sonicated for 30 min and filtered through 0.20 µm PTFE syringe filters. The filtered mixture was then injected into an Agilent 1200 series HPLC system, with a 0.05 M LiBr in DMF mobile phase, flowing at 0.6 mL/min. A stationary phase of one HT5 Styragel (WAT045945, Waters, Milford, MA, USA) followed by one Polargel-L (PL1117-6830, Agilent, Santa Clara, CA, USA) was used for separation, in conjunction with an Optilab-WREX-08 differential refractometer and a Wyatt Technology DAWN MALS (Santa Barbara, CA, USA) instrument (with filtered detectors) used for detection.

The ash content in the lignin was determined via thermogravimetric analysis (TGA). A total of ~10 mg of lignin was placed in a platinum holder of a thermogravimetric analyzer (Q5000, TA Instruments, New Castle, DE, USA), heated to 100 °C, held for 15 min, and then heated to 800 °C. The purge gas was air, and the heating ramp was 10 °C/min. The lignin ash content was defined as W/W_0_, where W_0_ is the initial lignin mass and W is the remaining mass of the sample at the end of the run.

The metal contents of the lignin and lignin fractions were measured via Inductively Coupled Plasma Atomic Emission Spectroscopy (ICP-AES, model ACROS, Spectro Analytical Instruments, Kleve, Germany). Before measurement, 100 mg dried lignin was digested in 5 mL concentrated nitric acid at 25 °C for 30 min and then further digested by heating to 125 °C for 90 min, followed by adding 3 mL hydrogen peroxide (H_2_O_2_) and heating at 125 °C for 60 min. Afterward, 3 mL additional H_2_O_2_ was added, and the sample was kept heated at 125 °C for 60 min. Finally, the sample was air dried at 200 °C for 1 h and the dried sample was diluted in 10 mL 1.6 M nitric acid and another 50 mL deionized water after cooling. The resulting liquid was transferred to the ICP tube for detection.

### 3.3. Activated Carbon Synthesis

A total of 1.8 g of lignin was dried in a vacuum oven at 60 °C for 12 h, and then sieved through a 60-mesh sieve. The lignin was mixed with ZnCl_2_ solution at a ratio of 2.5:1 by weight (ZnCl_2_ anhydrous:lignin) under stirring at 350 RPM for 24 h. The ZnCl_2_ solution was made by anhydrous ZnCl_2_ (98+%, Alfa Aesar, Haverhill, MA, USA) and enough DI water to obtain a ratio of 2.0 mL water per gram of total solids (ZnCl_2_ and lignin). The lignin–ZnCl_2_ mixture was then dried by rotary evaporation and by vacuum oven at 110 °C for 24 h. The dried mixture was packed in graphite foil and placed in the center of a horizontal quartz tube in an electric furnace (Lindberg/Blue M, Thermo Scientific, Waltham, MA, USA). The tube was purged with high-purity (+99.99%) nitrogen (N_2_) at a flow rate of 1000 cm^3^/min for 30 min and then the N_2_ flow rate was adjusted to 300 cm^3^/min for carbonization. The lignin was heated at 10 °C/min to 500 °C and held for 1 h. After cooling to an ambient temperature, the AC was washed with 150 mL 3 M hydrochloric acid (HCl) for 1 h under stirring at 350 RPM. The HCl acid was filtered (Nylon filter, 0.45 µm, Sigma Aldrich, St. Louis, MO, USA) and the AC was then rinsed by 60 °C DI water and filtered repeatedly until the conductivity of the washing water was near the conductivity of the DI water (~0.6 µS/cm). The AC was finally dried in vacuum at 110 °C for 24 h.

### 3.4. Activated Carbon Characterization

N_2_ adsorption and desorption isotherms of AC were measured at 77 K by using an automated gas sorption analyzer (Autosorb iQ, Quantachrome Instruments, Boynton Beach, FL, USA), after samples of approximately 150 mg were degassed at 250 °C for 7.5 h under vacuum. The specific surface area, reported at the BET surface area, was determined by the Brunauer–Emmett–Teller (BET) method and the pore size distribution was determined using the density functional theory (DFT) method, which are universal methods for characterizing activated carbon. Pore distribution data were determined from the N_2_ desorption data. The total pore volume was determined by the amount of N_2_ adsorption expressed in liquid form and a relative pressure (P/P_0_) of approximately 0.95. The average pore width was calculated using the specific surface area and the total pore volume by the Gurwitch rule. According to the International Union of Pure and Applied Chemistry (IUPAC) classification, pores with widths below 2 nm are micropores, pores with widths between 2 nm and 50 nm are mesopores, and pores with widths greater than 50 nm are macropores [51].

The aqueous adsorption capacity of AC was determined by the iodine (I_2_) number and methylene blue (MEB) value. A total of 60 mg of AC and 20 mg of AC were, respectively, mixed with 20 mL 0.1 M standard I_2_ solution and 20 mL 600 mg/L MEB solution under stirring at 125 RPM for 24 h. The mixtures were centrifuged, and clear I_2_ and MEB solutions were decanted. The clear I_2_ solution was titrated by 0.025 M sodium thiosulfate (Na_2_S_2_O_3_) and the concentration of the clear MEB solution was measured by an ultraviolet–visible (UV–Vis) spectrophotometer (Agilent BioTek Microplate Readers, Agilent) compared against a calibration curve.

### 3.5. Carbon Fractional Conversion

The carbon fractions in lignin and AC were determined by elemental analysis of combustion products using automatic analyzers in Atlantic Microlab, Inc. (Atlanta, GA, USA). Before the combustion, the lignin was vacuum dried (0.015 mmHg) at 120 °C for 2 h and the AC was vacuum dried (0.015 mmHg) at 250 °C for 4 h before combustion. The carbon fractional conversion is defined as the mass of carbon in AC divided by the mass of carbon in the lignin. The overall yield is defined as the AC mass divided by the lignin mass.

## 4. Conclusions

Using ZnCl_2_ activation with low-temperature carbonization condition, AC was synthesized from lignin with varying MW controlled by the ALPHA process. We found that the surface area and total pore volume of AC increase as the lignin MW increases. The AC derived from lignin with the highest MW of 44,200 Da possessed the highest surface area of 1830 m^2^/g and the highest total pore volume of 1.27 cm^3^/g. When lignin is in the low-MW range, pore enlargement is more significant with the decrease in lignin MW, leading to an expansion in the average pore width of AC. Moreover, we observed that the overall yield and carbon fractional conversion from lignin to AC are not influenced by the lignin MW, but rather depend on the ash content of the sample. Regarding the impact of ash on AC properties, the surface area and total pore volume of AC are lower when synthesized from lignin samples with a higher ash content. A higher ash content also promotes pore enlargement. The understanding of these effects of lignin properties on AC properties is instructive for the optimization of industrial-scale AC production from lignin. This knowledge can substantially contribute to achieving the highest surface area for AC, all while maintaining the lowest possible production cost. With knowledge of how some features of lignin precursors impact pore development in activated carbon, future research will need to address the differences in the chemical composition of lignin, such as variation in oxygen content, from different sources. Furthermore, the activation and carbonization conditions applied here were optimized for biomass that contain more than just lignin, so it will be important to adjust these conditions and even explore other synthesis methods in order to understand how to best utilize lignin-rich byproduct streams.

## Data Availability

Data are contained within the article.
